# Rooibos Flavonoids Inhibit the Activity of Key Adrenal Steroidogenic Enzymes, Modulating Steroid Hormone Levels in H295R Cells

**DOI:** 10.3390/molecules19033681

**Published:** 2014-03-24

**Authors:** Lindie Schloms, Amanda C. Swart

**Affiliations:** Department of Biochemistry, Stellenbosch University, Private Bag X1, Matieland 7602, South Africa; E-Mail: schloms@sun.ac.za

**Keywords:** *Aspalathus linearis*, rooibos polyphenols, adrenal steroidogenesis, cytochrome P450 enzymes, UPLC-MS/MS, adrenal H295R cells, cortisol, metabolic disorder, structure-activity relationship, stress

## Abstract

Major rooibos flavonoids—dihydrochalcones, aspalathin and nothofagin, flavones—orientin and vitexin, and a flavonol, rutin, were investigated to determine their influence on the activity of adrenal steroidogenic enzymes, 3β-hydroxysteroid dehydrogenase (3βHSD2) and cytochrome P450 (P450) enzymes, P450 17α-hydroxylase/17,20-lyase (CYP17A1), P450 21-hydroxylase (CYP21A2) and P450 11β-hydroxylase (CYP11B1). All the flavonoids inhibited 3βHSD2 and CYP17A1 significantly, while the inhibition of downstream enzymes, CYP21A2 and CYP11B1, was both substrate and flavonoid specific. The dihydrochalcones inhibited the activity of CYP21A2, but not that of CYP11B1. Although rutin, orientin and vitexin inhibited deoxycortisol conversion by CYP11B1 significantly, inhibition of deoxycorticosterone was <20%. These three flavonoids were unable to inhibit CYP21A2, with negligible inhibition of deoxycortisol biosynthesis only. Rooibos inhibited substrate conversion by CYP17A1 and CYP21A2, while the inhibition of other enzyme activities was <20%. In H295R cells, rutin had the greatest inhibitory effect on steroid production upon forskolin stimulation, reducing total steroid output 2.3-fold, while no effect was detected under basal conditions. Nothofagin and vitexin had a greater inhibitory effect on overall steroid production compared to aspalathin and orientin, respectively. The latter compounds contain two hydroxyl groups on the B ring, while nothofagin and vitexin contain a single hydroxyl group. In addition, all of the flavonoids are glycosylated, albeit at different positions—dihydrochalcones at C3' and flavones at C8 on ring A, while rutin, a larger molecule, has a rutinosyl moiety at C3 on ring C. Structural differences regarding the number and position of hydroxyl and glucose moieties as well as structural flexibility could indicate different mechanisms by which these flavonoids influence the activity of adrenal steroidogenic enzymes.

## 1. Introduction

Flavonoids are a diverse group of plant-derived polyphenols that occur naturally in fruits, vegetables, teas and herbs. These secondary plant metabolites have been shown to exhibit potent anti-oxidant activities, scavenging reactive radicals that cause cellular damage associated with many diseases and clinical conditions. Flavonoids, often referred to as phytoestrogens due to their structural similarity to estrogen and ability to bind the estrogen receptor, may have implications in various clinical conditions and hormone-dependent cancers [1,2]. Although flavonoid polyphenols are generally associated with beneficial health properties, it has been suggested that when consumed in high dosages, flavonoids may act as pro-oxidants and mutagens resulting in cytotoxicity. One such flavonoid, apigenin, a common dietary flavone which exhibits anti-inflammatory, anti-oxidant and anti-carcinogenic properties, was recently shown to induce oxidative stress, causing liver damage in Swiss mice following administration of high dosages of the compound [3,4]. Flavonoids have also been shown to modulate key enzymes in adrenal steroidogenesis, affecting steroid hormone biosynthesis in the mineralocorticoid, glucocorticoid and adrenal androgen pathways [5,6,7,8]. Abnormal adrenal steroid hormone levels impact on human health, leading to a broad spectrum of clinical conditions. Chronically elevated cortisol levels have, for example, been associated with metabolic disorders such as visceral obesity, insulin resistance, hypertension, cardiovascular disease and type 2 diabetes [9]. Clinical strategies employed in the treatment of diseases associated with endocrine disorders and cancers include selective inhibitors of either the enzymes catalysing adrenal steroid biosynthesis and metabolism or antagonist activity at steroid receptor level [10]. Diets supplemented with flavonoid-rich plant based foods and beverages may influence the endocrine system and impact on metabolic diseases.

Rooibos, a polyphenol-rich herbal tea prepared as an infusion from the stems and leaves of the plant *Aspalathus linearis*, has traditionally been used to aid in the alleviation of sleeplessness, anxiety and nervous tension, ailments related to stress and physiological conditions generally associated with high cortisol levels. Our previous studies showed that rooibos and specific flavonoids inhibit cytochrome P450 (P450) enzymes and cortisol production [8,11].

*In vitro* studies in COS-1 cells have shown that unfermented rooibos extract as well as the two rare dihydrochalcones, aspalathin and nothofagin, significantly inhibited P450 17α-hydroxylase/17,20 lyase (CYP17A1) and P450 21-hydroxylase (CYP21A2), while also significantly reducing the levels of cortisol in forskolin stimulated adrenal H295R cells. Even though aspalathin and nothofagin are the two most abundant flavonoids in unfermented rooibos, they did not in all cases reflect the same inhibitory effect on steroid levels in H295R cells as brought about by the rooibos extract, suggesting that other compounds contribute to the overall effect of rooibos on adrenal steroidogenesis [8].

In the present study we continued our investigation into the influence of rooibos and polyphenolic compounds, belonging to three distinct classes of flavonoids, on adrenal steroidogenic enzymes. The five major rooibos flavonoids used in this study included the dihydrochalcones, aspalathin and nothofagin, their flavone analogues, orientin and vitexin, as well as the flavanol, rutin (Table 1). Due to the structural differences of the flavonoids, these compounds may interact differently with the steroidogenic enzymes thus affecting steroid hormone biosynthesis in the adrenal. We therefore determined the inhibitory effect of rooibos flavonoids on enzymes that play a key role in adrenal steroidogenesis, 3β-hydroxysteroid dehydrogenase (3βHSD2), CYP17A1, CYP21A2 and 11β-hydroxylase (CYP11B1), as well as on overall steroid hormone production in the human adrenal H295R cell line under both basal and forskolin stimulated conditions. 

**Table 1:** Chemical structures of the major rooibos flavonoids.

Structure	Compound	Substitution
Dihydrochalcones 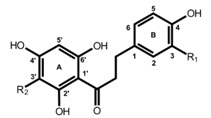	Aspalathin	R_1_=OH, R_2_=C-β-d-glucopyranosyl
	Nothofagin	R_1_=H, R_2_=C-β-d-glucopyranosyl
Flavones 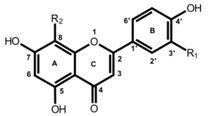	Orientin	R_1_=OH, R_2_=C-β-d-glucopyranosyl
	Vitexin	R_1_=H, R_2_=C-β-d-glucopyranosyl
Flavonol 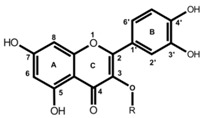	Rutin	R=rutinosyl

## 2. Results and Discussion

It has been widely reported that polyphenols exhibit a diverse range of beneficial biological effects, and as such, their consumption as part of the daily diet cannot be ignored. Although teas and herbal infusions contribute to the daily intake of dietary flavonoids, the pharmacological actions of these extracts cannot directly be attributed to single compounds within these beverages, unless identified and assayed in isolation. Flavonoids modulate steroid hormone biosynthesis and metabolism due to their interaction with steroidogenic enzymes, either inhibiting or stimulating specific enzymes [7,8]. We recently showed that unfermented rooibos extract influenced the steroid flux in the mineralocorticoid, glucocorticoid and androgen precursor pathways in human adrenal H295R cells under both basal and forskolin stimulated conditions [8]. While the effect of flavonoid compounds on the flux through the adrenal steroidogenic pathways can be determined, the influence on specific enzymes in these pathways is less obvious due to upstream inhibition of steroid intermediates. In the present study, we assayed the effect of flavonoid compounds on individual steroidogenic enzymes as well as on steroid metabolite levels in H295R cells using ultra performance liquid chromatography/tandem mass spectrometry (UPLC-MS/MS).

### 2.1. The Influence of Rooibos and Selected Flavonoid Compounds on Adrenal Steroidogenic Enzymes Expressed in Non-Steroidogenic COS-1 Cells

**Scheme 1:**
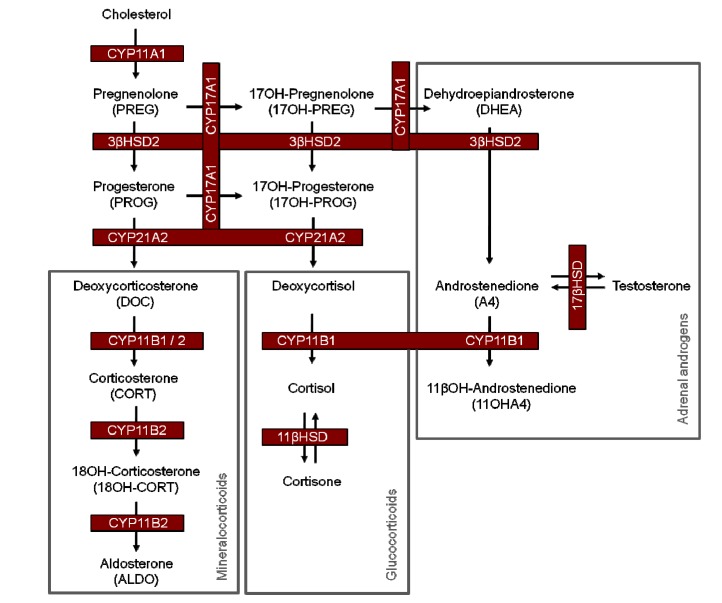
Steroid hormones produced in human adrenal cells.

CYP17A1 plays a central role at the branch point in adrenal steroid biosynthesis, and together with 3βHSD2, determines the shunt of steroid metabolites in the glucocorticoid, mineralocorticoid and adrenal androgen pathways (Scheme 1).

**Figure 1:**
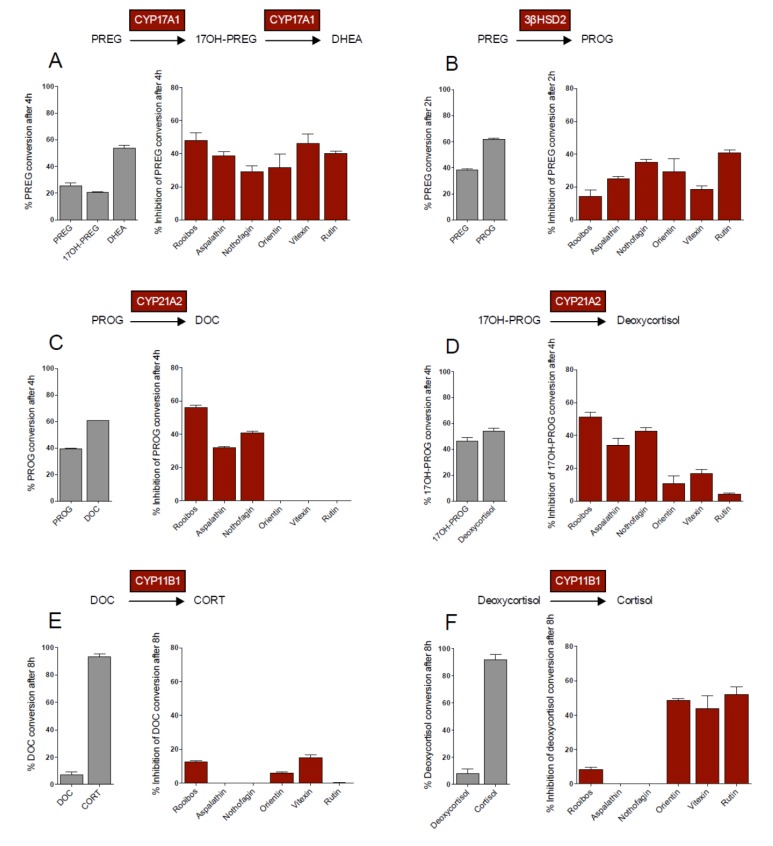
Substrate conversion (1 µM) in transiently transfected COS-1 cells in the absence and presence of rooibos extract (4.3 mg/mL) and selected flavonoids (10 µM). CYP17A1 and CYP21A2 data adapted from [8].

CYP17A1 catalyses the 17α-hydroxylation of pregnenolone (PREG), yielding 17OH-pregnenolone (17OH-PREG), which is in turn converted to dehydroepiandrosterone (DHEA). These three metabolites are substrates for 3βHSD2, which catalyzes their conversion to progesterone (PROG), 17OH-progesterone (17OH-PROG), and androstenedione (A4), respectively, thereby shunting metabolites into the respective pathways. In addition, CYP17A1 also catalyses the conversion of PROG to 17OH-PROG and 16OH-progesterone (16OH-PROG), with the latter not being further metabolized in the adrenal. PREG conversion by CYP17A1 was inhibited by all the flavonoid compounds assayed, with the compounds exhibiting similar inhibitory effects (Figure 1A). Although PREG conversion by 3βHSD2 was also inhibited by all the flavonoids assayed, rutin exhibited the greatest inhibitory effect, while vitexin had the lowest inhibitory effect, 2-fold lower than that of rutin (Figure 1B).

CYP21A2 catalyzes the conversion of PROG and 17OH-PROG to DOC and deoxycortisol, respectively. In contrast to CYP17A1 and 3βHSD2, only the dihydrochalcones inhibited the activity of CYP21A2 significantly, with the flavones and rutin not influencing the conversion of PROG by CYP21A2 (Figure 1C). In the conversion of 17OH-PROG, however, these compounds displayed inhibitory effects below 20% (Figure 1D). Neither aspalathin nor nothofagin inhibited the conversion of DOC and deoxycortisol by CYP11B1 to their corresponding products, CORT and cortisol, respectively. The main structural difference between the dihydrochalcones and the other flavonoids assayed in this study is the structural flexibility of the molecules and the glucose moiety at C3' on ring A of the dihydrochalcones, which could interfere with the binding of the compounds to CYP11B1. Inhibition of CYP11B1 by vitexin, orientin and rutin, however, was substrate specific. While inhibition of DOC conversion was negligible (Figure 1E), inhibition of deoxycortisol conversion was significantly higher in the presence of rutin, orientin and vitexin (Figure 1F). The higher binding affinity of CYP11B1 for DOC compared to deoxycortisol may account for the negligible inhibition of the flavonoids on the conversion of DOC. CYP11B1 has been shown to exhibit a lower Km for DOC and its catalytic conversion is also characterized by a lower K_cat_, 2-fold less than that of deoxycortisol conversion, clearly indicating that a single hydroxyl group influences the enzyme’s affinity for its substrate, as well as the substrate turnover. It was suggested that the presence of the C17 hydroxyl group of deoxycortisol obstructs the entrance of deoxycortisol into the enzyme’s active pocket [12].

While the rooibos extract exhibited the greatest inhibitory effect on CYP17A1 and CYP21A2, inhibition of the other enzymes was negligible. The inhibition of PREG conversion by CYP17A1 (Figure 1A) as well as the conversion of PROG (Figure 1C) and 17OH-PROG (Figure 1D) by CYP21A2 was similar (±50%) in the presence of the extract. However, the inhibitory effect of rooibos on PREG conversion by 3βHSD2 (Figure 1B) and DOC (Figure 1E) and deoxycortisol (Figure 1F) conversion by CYP11B1, was less than 20%. The inhibitory effects brought about by the individual flavonoid compounds (10 µM) was not reflected in the inhibitory effects of the whole extract in all instances, even though the flavonoid concentrations within the rooibos extract ranged between 27 µM and 1.4 mM. Although we assayed five of the major flavonoids present in rooibos, it is important to note that, to date, 46 flavonoid compounds have been identified in rooibos [13,14] which may possibly contribute to the data obtained in the presence of the extract. From the data it is clear, however, that the rooibos extract preferentially inhibits CYP17A1 and CYP21A2, two key enzymes at the branch point of adrenal steroidogenesis.

### 2.2. The Influence of Selected Flavonoids on Steroid Metabolism in Adrenal H295R Cells under Basal and Forskolin Stimulated Conditions

In H295R cells, the dihydrochalcones and the flavones decreased the total steroid output under both basal and stimulated conditions, while rutin’s effect was only detected in the presence of forskolin. Forskolin mimics the effects of ACTH by activating cAMP pathways in adrenal cells [15], stimulating steroidogenic enzymes and increasing steroid production. As expected, forskolin significantly increased the total steroid output to 11.1 µM (2.8 fold), with the greatest inhibition being detected in the presence of rutin (Table 2). Both aspalathin and nothofagin significantly reduced basal 17OH-PROG and 16OH-PROG levels due to the inhibition of CYP17A1 and 3βHSD2. Nothofagin also reduced deoxycortisol levels under basal conditions, possibly due to inhibition of CYP21A2 or due to lower levels of 17OH-PROG being available as the precursor substrate. Both dihydrochalcones reduced A4 levels significantly under basal and forskolin stimulated conditions, indicative of 3βHSD2 inhibition. Interestingly, while nothofagin reduced basal levels of 11β-hydroxyandrostenedione (11OHA4), the product of A4, it did not reduce basal production of cortisol, which is catalysed by the same enzyme, CYP11B1. Although this may suggest possible inhibition of CYP11B1, neither aspalathin nor nothofagin were shown to inhibit the enzyme at 10 µM in COS-1 cells, suggesting that the reduction in 11OHA4 was due to upstream inhibition. Under stimulated conditions, the production of both cortisol and 11OHA4 was inhibited in the presence of aspalathin and nothofagin, indicating upstream inhibition, resulting in reduced levels of precursor steroid metabolites. It should, however, also be noted that when comparing inhibitory effects of the flavonoid compounds under basal and stimulated conditions, the expression levels of steroidogenic enzymes are altered when cells are stimulated with forskolin, which would in turn alter steroid metabolite/precursor levels. From these results it is clear that the inhibitory effects of both dihydrochalcones are very similar, however, the inhibitory effect of nothofagin was notably greater than that of aspalathin.

**Table 2:** Steroid metabolites produced in adrenal H295R cells under basal and forskolin (10 µM) stimulated conditions in the absence and presence of selected flavonoids (10 µM) after 48 h.

Steroid metabolites	Basal			+ Aspalathin ^a^			+ Nothofagin ^a^			+ Orientin			+ Vitexin			+ Rutin ^b^		
	Total ± SEM (nM)			Fold change			Fold change			Fold change			Fold change			Fold change		
PREG	35.2	±	2.2										↑	1.4	**			
PROG	5.8	±	1.7															
DOC	107.4	±	13.0							↑	1.2	*	↑	1.2	**			
CORT	241.1	±	31.6							↑	1.2	*						
18OH-CORT	9.3	±	1.3							↑	2.6	*						
ALDO	2.3	±	0.5															
11-DHC	9.9	±	0.3															
16OH-PROG	66.4	±	10.3	↓	2.3	***	↓	3.6	***	↓	1.2	***	↓	1.8	***			
17OH-PROG	56.8	±	6.3	↓	3.6	**	↓	4.8	**				↓	1.4	*			
Deoxycortisol	1741.0	±	234.1				↓	2.3	*				↓	1.3	***			
Cortisol	670.2	±	39.3										↓	1.3	**			
Cortisone	6.6	±	2.2							↓	1.5	***	↓	1.8	***			
A4	806.5	±	115.6	↓	2.4	**	↓	3.5	***	↓	1.1	*	↓	1.5	***			
11OHA4	90.1	±	11.1				↓	3.0	*				↓	1.5	***			
Testosterone	39.22	±	3.60	↓	4.3	***	↓	7.1	***	↓	1.3	**	↓	2.5	***			
Total steroid (nM)	3947.0			↓	1.6		↓	2.0	*	↓	1.0		↓	1.3	**	↓	1.0	
**Steroid metabolites**	**Forskolin**			**+ Aspalathin ^a^**			**+ Nothofagin ^a^**			**+ Orientin**			**+ Vitexin**			**+ Rutin ^b^**		
	**Total ± SEM (nM)**			**Fold change**			**Fold change**			**Fold change**			**Fold change**			**Fold change**		
PREG	47.5	±	3.4							↑	1.4	**	↑	1.3	**			
PROG	5.1	±	0.5							↑	1.4	**	↑	1.5	**			
DOC	343.2	±	61.1							↓	1.2	*	↓	1.1	*			
CORT	2062.0	±	170.5										↓	1.3	**	↓	1.9	*
18OH-CORT	104.8	±	15.1													↓	2.0	**
ALDO	27.4	±	3.4							↓	1.1	***	↓	1.4	*			
11-DHC	11.2	±	1.4															
16OH-PROG	66.0	±	4.7										↓	1.4	**			
17OH-PROG	36.0	±	2.4													↓	3.1	*
Deoxycortisol	2757.0	±	393.1										↓	1.3	*	↓	2.0	**
Cortisol	3793.0	±	285.1	↓	1.3	***	↓	1.7	***	↓	1.3	***	↓	1.8	***	↓	3.0	***
Cortisone	11.6	±	2.7				↓	2.6	*									
A4	1402.0	±	180.0	↓	1.7	***	↓	2.0	***	↓	1.4	***	↓	1.5	***	↓	4.4	***
11OHA4	388.3	±	16.3	↓	1.3	**	↓	2.5	***	↓	1.5	***	↓	1.7	***	↓	6.9	***
Testosterone	50.5	±	6.1	↓	1.7	***	↓	2.4	***	↓	1.7	***	↓	2.3	***	↓	5.8	***
Total steroid (nM)	11,141.0			↓	1.4	*	↓	1.4	*	↓	1.1		↓	1.3	**	↓	2.3	**

* *p* < 0.05, ** *p* < 0.01, *** *p* < 0.001; ^a^ Adapted from [8]; ^b^ Adapted from [11]; ↑ (increase) or ↓ (decrease) in steroid levels (nM) under basal / forskolin stimulated conditions in the presence of flavonoids.

Orientin, the flavone analogue of aspalathin, significantly increased the steroid flux in the mineralocorticoid pathway, increasing basal levels of DOC, CORT and 18OH-CORT, suggesting that inhibition of CYP17A1 resulted in more metabolites being channeled into the mineralocorticoid pathway. The data obtained clearly shows that the flavones inhibit CYP17A1, since PREG and PROG levels increased, while 16OH-PROG and 17OH-PROG levels decreased (Table 2).

The inhibitory profile of vitexin was very similar to that of orientin, however, vitexin exhibited a greater inhibitory effect, reducing the total steroid output 1.3-fold under both basal and stimulated conditions, while the effect of orientin on total steroid production was negligible. Interestingly, under stimulated conditions, during which aldosterone synthase (CYP11B2) expression is upregulated [16], the effect of vitexin on the mineralocorticoid pathway was much more prominent compared to orientin, with a greater reduction in ALDO levels being observed, suggesting inhibition of either CYP11B2 or upstream enzymes. Under both basal and stimulated conditions, vitexin also had a much greater effect on the glucocorticoid pathway compared to orientin, reducing the levels of cortisol and its precursors significantly. In COS-1 cells, rutin as well as the flavones inhibited the biosynthesis of cortisol by CYP11B1, while the dihydrochalcones did not, suggesting that the reduced cortisol levels observed in H295R cells in the presence of aspalathin and nothofagin may be indicative of upstream inhibition only.

While the dihydrochalcones and their flavone analogues reduced basal testosterone levels significantly, it was the former compounds which had the greatest effect, suggesting inhibition of 17βHSD. However, the adrenal is not the primary site for 17βHSD expression, as is also the case for 11βHSD2, which catalyses the formation of cortisone and 11-dehydrocorticosterone (11-DHC). The detected levels of these metabolites were very low, with the flavones reducing cortisone levels under basal conditions, while under stimulated conditions, it was inhibited in the presence of nothofagin only. Although 17βHSD and 11βHSD2 are expressed at very low levels in the adrenal [17], it would appear that these hydroxysteroid dehydrogenases, together with 3βHSD2, are sensitive to the flavonoid compounds present in Rooibos.

In a recent pharmacophore-based virtual screening study investigating inhibitors of 17βHSD type 3 and 5, key enzymes involved in adrenal androgen production, it was shown that the ligand binding domain of these enzymes is able to accommodate structurally highly diverse ligands which bind to different regions of the active site. An enlargement of the binding cavity was observed in the crystal structure of 17βHSD3/5 when rutin was bound to these enzymes. Furthermore, the X-ray structure of rutin bound to 17βHSD5 showed that water molecules formed a hydrogen bonding network with rutin bound at the base of the ligand binding domain [18].

Rutin, one of the more stable flavonoid compounds in both fermented and unfermented Rooibos, had no significant effect on any of the steroid hormones under basal conditions. However, upon forskolin stimulation, rutin had the greatest inhibitory effect of all the flavonoid compounds assayed, reducing the total steroid output 2.3-fold. In the mineralocorticoid pathway, CORT and 18OH-CORT levels were significantly lower, suggesting inhibition of CYP11B2. In the glucocorticoid pathway, significant reductions in the levels of 17OH-PROG, deoxycortisol and cortisol indicate inhibition of CYP17A1 and CYP11B1, as was also shown in COS-1 cells (Figure 1). Upstream inhibition also contributed to the reduced deoxycortisol levels, since rutin did not inhibit CYP21A2. In the adrenal androgen precursor pathway, rutin reduced the levels of A4, testosterone and 11OHA4, suggesting inhibition of CYP17A1 and/or 3βHSD2.

The data obtained in the H295R cell line did not in all cases reflect results obtained in COS-1 cells. The expression levels of the individual enzymes assayed in COS-1 cells are comparable, as the cells were transiently transfected with equal concentrations of the appropriate cDNA. Furthermore, the conversion of substrates in COS-1 cells allowed assays to be conducted under very specific conditions. A fixed substrate concentration and no competition from other enzymes for the same substrates, as is the case in the adrenal H295R cell model, allowed us to investigate the effect of flavonoids on the catalytic activity of specific steroidogenic enzymes. In contrast, enzymes are expressed at different levels in adrenal H295R cells and vary from enzyme to enzyme under both basal and forskolin stimulated conditions, with specific enzymes being upregulated when cells are stimulated. It was recently shown that under basal conditions in H295R cells, CYP11A1 is expressed at the highest levels, followed by CYP21A2, CYP17A1, SULT2A1, 3βHSD2, CYP11B2 and CYP11B1, while under forskolin stimulated conditions, CYP17A1 is expressed at the highest levels, followed by CYP21A2, CYP11A1, 3βHSD2, SULT2A1, CYP11B2, and CYP11B1 [16].

Overall, from these results it is clear that orientin and vitexin had the greatest effect on the mineralocorticoid pathway under both basal and forskolin stimulated conditions. The dihydrochalcones had no significant effect on any of the metabolites in the mineralocorticoid pathway, while rutin’s effect was only detected under stimulated conditions, with significantly reduced levels of ALDO precursors, CORT and 18OH-CORT. The influence of rutin was most evident in the glucocorticoid and adrenal androgen pathways under stimulated conditions, with the other flavonoids decreasing metabolites under both basal and stimulated conditions in these pathways.

### 2.3. Structure Activity Analyses

Analyses regarding the influence of structural differences of flavonoid compounds on their ability to inhibit steroidogenic enzymes are hampered due to the uncertainty of the manner in which these compounds would interact with the active site of these enzymes. As the type of inhibition of the steroidogenic cytochromes P450 by the flavonoids is not known, it is possible that binding could also occur at sites other than the active pocket. Furthermore, molecular docking studies reporting on structural aspects of flavonoid compounds impacting on substrate inhibition in terms of steroidogenic enzymes are limited. It has, however, been shown by Ohno *et al.* that the isoflavone, diadzein, was able to competitively inhibit the binding of DHEA and PROG to 3βHSD2 and CYP21A2, respectively. The authors suggested, having assayed a range of flavone compounds, that it was the hydroxyl groups at C6 on ring A and C4' on ring B which played an important role in the inhibition of steroidogenic P450 enzymes. They showed, in cAMP-stimulated H295R cells, at a concentration of 12.5 µM, that 6-hydroxyflavone, 4'-hydroxyflavone and apigenin, which contains hydroxyl groups at C5, C7 and C4', inhibited cortisol production significantly [7]. In the present study, vitexin, with a C4' hydroxyl group and orientin with C3' and C4' hydroxyl groups, both also containing a glucose moiety at C8, significantly inhibited cortisol biosynthesis at a concentration of 10 µM. In COS-1 cells, it was the flavones and rutin that inhibited CYP11B1 significantly, while the dihydrochalcones did not inhibit the activity of CYP11B1 towards DOC or deoxycortisol. It is interesting to note that the flavones as well as rutin, containing glucose moieties on ring A and ring C respectively, showed negligible inhibition of CYP21A2. In contrast, while aspalathin and nothofagin significantly inhibited substrate conversion by CYP21A2, these compounds had no influence on the conversion of either DOC or deoxycortisol by CYP11B1, possibly due to the glucosyl moiety at C3' on ring A. Our data thus far suggests that a glucosyl moiety at this position may prevent compounds inhibiting the catalytic activity of CYP11B1, while the C8 glucosyl and C3 rutinosyl moieties on the pyran ring of the other flavonoids do not. Substitutions at C3 and C4 (dihydrochalcones) and C3' and C4' (flavones and flavonol) on the B ring may, however, also play a role. The type of inhibition that these compounds elicit need to be determined, while homology models may pinpoint the effect of functional groups on the binding of the compounds in the active pocket of the relevant enzymes.

In a more recent study by Hasegawa *et al.* [5], it was also shown in H295R cells that a range of flavones with hydroxyl groups at positions C4' or C3' and C4' on ring B significantly reduced the levels of DOC and A4, suggesting inhibition of 3βHSD2, while apigenin was more potent than the other polyphenols assayed, increasing the levels of PREG and 17OH-PROG, suggesting inhibition of CYP17A1, CYP21A2 and 3βHSD2. Apigenin was also shown to downregulate the expression levels of CYP17A1, CYP21A2 and 3βHSD2 mRNA significantly [5].

From our data it appears that the enzymes 3βHSD2 and CYP17A1 are most susceptible to inhibition by the rooibos flavonoids assayed in this study. The natural substrates for these two enzymes, PREG, 17OH-PREG and DHEA contains a hydroxyl group on C3 of the steroid backbone structure involved with substrate binding in the active pocket [19,20]. It is possible that the hydroxyl group on position C4' of the flavonoid is involved in the binding of these compounds to the active pocket of steroidogenic P450 enzymes, with hydrogen bonds stabilizing the orientation of flavonoid compounds in the active site. However, since limited data is available on the molecular docking of flavonoids, no conclusions can be drawn regarding the orientation of these compounds within the active pocket. In addition, as the mechanism of inhibition is uncertain, the binding of these compounds to the same site that the substrate would occupy cannot be assumed. A recent study by Androutsopoulos *et al.* [21], investigating the binding mode of selected flavonoids to the heme group using the CYP1A2 crystal structure as a template, predicted that flavonoids bind in the active pocket with the B-ring orientated towards the heme group. In an earlier study, Shimada *et al.* [22] investigated the inhibition of a range flavonoid compounds towards five human P450 enzymes by assessing inhibitory activity together with molecular docking studies. These studies clearly showed that the position and number of hydroxy and methoxy groups impacted on the orientation of the compounds in the active pocket of the different enzymes. It was shown that ring B of the compounds was not, in all cases, oriented towards the heme group and that hydroxy/methoxy substitutions influenced positioning of the flavonoid compounds and thus also affected the mechanisms of inhibition for the enzymes assayed.

The compounds assayed in this study all contained glucose moieties on either ring A or C, which would, if bound in the active site of the enzyme, affect the orientation of the molecule, impacting on the mechanism of inhibition. It would seem that while a hydroxy group on B ring plays a role in flavonoid inhibitory activity, a second hydroxy group does not significantly affect inhibition or contribute towards increasing inhibition, possibly due to rotation between rings A and B resulting in a degree of flexibility. It should, however, also be noted that aspalathin and nothofagin have a more flexible structure compared to rutin, orientin and vitexin. It is interesting to note that the dihydrochalcones inhibit the catalytic activity of CYP21A2 towards both substrates, while exhibiting no inhibitory effect on CYP11B1, regardless of the substrate. In contrast, the inhibition of the activity of these enzymes towards their substrates differs in the presence of flavone/flavonol compounds, with the inhibitory effect of these compounds being greater when the substrate is hydroxylated at the C17 position.

## 3. Experimental

### 3.1. Reagents and Instruments

Unfermented rooibos plant material was provided by the South African Rooibos Council (Rooibos LTD-BPK, Clanwilliam, South Africa). Aspalathin and nothofagin were obtained from Prof. W.C.A. Gelderblom (Medical Research Council, Western Cape, South Africa). Orientin and vitexin were purchased from Extrasynthese (Genay Cedex, France). Rutin, steroid metabolites, forskolin, Dulbecco’s modified Eagle’s Medium (DMEM) and an MTT assay kit were purchased from Sigma-Aldrich (St. Louis, MO, USA). Nucleobond^®^ AX plasmid preparation kits were purchased from Machery-Nagel (Duren, Germany). Mirus TransIT^®^-LT1 transfection reagent was purchased from Mirus Bio Corporation (Madison, WI, USA). Penicillin–streptomycin, fetal calf serum and trypsin-EDTA were obtained from Gibco BRL (Gaithersburg, MD, USA). Deuterated cortisol (9,11,12,12-D4-cortisol) was purchased from Cambridge isotopes (Andover, MA, USA). DMEM/F_12_ and gentamicin were purchased from Invitrogen/Gibco (Grand Island, New York, USA). Cosmic calf serum was supplied by HyClone^®^, Thermo Scientific (South Logan, Utah, USA). A bicinchoninic acid (BCA) protein determination kit was purchased from Pierce (Rockford, IL, USA). The UPLC BEH C18 column was purchased from Waters (Milford, MA, USA). The Kinetex PFP column was obtained from Phenomenex (Torrance, CA, USA). All chemicals were of the highest quality and supplied by reputable scientific suppliers.

### 3.2. Substrate Conversion Assays in Transiently Transfected COS-1 Cells

COS-1 cells were grown at 37 °C and 5% CO_2_ in DMEM containing 0.9 g/L glucose, 0.12% NaHCO_3_, 10% fetal calf serum and 1% penicillin streptomycin. The cells were plated into 12 well dishes (1 × 10^5^ cells/mL, 1 mL/well), 24 h prior to transfection. Cells were transiently transfected with 0.5 μg cDNA (baboon CYP17A1/pCIneo, baboon 3βHSD2/pCIneo, baboon CYP21A2/pCIneo) and 1.5 µL *Mirus TransIT^®^*- LT1 transfection reagent according to the manufacturer’s instructions. Co-transfections with baboon CYP11B1/pTarget and human ADX/pCIneo cDNA were carried out using 0.25 µg of each plasmid. Control transfection reactions were performed using the pCIneo vector containing no cDNA insert. Cells were incubated for 72 h prior to substrate addition. Substrate conversion (1 µM) was assayed in the absence and presence of aspalathin, nothofagin, rutin, orientin and vitexin, assayed at 10 µM, and unfermented rooibos extract, assayed at 4.3 mg/mL. The methanolic extract of unfermented rooibos plant material used in this study was prepared and analysed as previously described [8,11]. At specific time intervals, 500 µL aliquots were removed and the steroids were extracted using a 10:1 volume of dichloromethane to culture medium. The mixture was vortexed for 30 s, centrifuged at 500 ×*g* for 5 min and the medium removed via aspiration. The dichloromethane phase, containing the steroid metabolites, was dried under N_2_, resuspended in 150 μL methanol and stored at −20 °C prior to analyses. 

### 3.3. Steroid Metabolism in Adrenal H295R Cells

H295R cells were grown at 37 °C and 5% CO_2_ in DMEM/F_12_, supplemented with l-glutamine, 15 mM HEPES, pyridoxine, 1.125 g NaHCO3/l, 1% penicillin streptomycin, 0.01% gentamicin, and 10% cosmic calf serum (growth medium). The cells were plated into 12 well dishes (4 × 10^5^ cells/mL, 1 mL/well) and incubated for 48 h. The medium was subsequently replaced with experimental medium (growth medium containing 0.1% cosmic calf serum) and cells were incubated for a further 12 h prior to substrate addition. Steroid metabolism was assayed in the presence of selected flavonoid compounds, aspalathin, nothofagin, rutin, orientin and vitexin (10 µM), under both basal and forskolin (10 µM) stimulated conditions. After 48 h, the medium (500 µL) was removed and 15 ng D4-cortisol added as an internal standard. The steroids were extracted as described in Section 3.2.

### 3.4. Cell Viability

COS-1 cells were plated out in a 96 well plate (100 µL, 1 × 10^5^ cells/mL) and incubated with selected flavonoid compounds (10 µM) for 8 h. H295R cells were plated out in a 96 well plate (100 µL, 4 × 10^5^ cells/mL) and incubated with selected flavonoids (10 µM) and forskolin (10 µM) for 48 h. Cell viability was assayed using an MTT toxicology assay kit according to the manufacturer’s instructions. Color-standardized controls (media containing either 1 mg/mL or 4.3 mg/mL Rooibos extract) were included to compensate for the possible interference of the Rooibos color with the assay. None of the abovementioned test compounds had a significant effect on COS-1 or H295R cell viability.

### 3.5. Separation and Quantification of Steroid Metabolites Using UPLC-MS/MS

All steroid metabolites were analysed and quantified using UPLC–MS/MS (ACQUITY UPLC, Waters, Milford, MA, USA). Steroid metabolites from conversion assays in COS-1 cells were separated using a Waters UPLC BEH C18 (2.1 mm × 50 mm, 1.7 µm) column, while steroid metabolites produced in H295R cells were separated using a Phenomenex UPLC Kinetex PFP (2.1 mm × 100 mm, 2.6 µm) column, as previously described [11]. A Xevo triple quadrupole mass spectrometer (Waters, Milford, USA) was used for quantitative mass spectrometric detection. All steroids were analysed in multiple reaction monitoring (MRM) mode using an electrospray in the positive ionization mode (ESI+). Gradients of the LC system and other relevant information including the mobile phases, flow rates, injection volumes, parent and daughter ions, cone (V) and collision (eV) voltages, retention times and the validation of the UPLC–MS/MS assays has been described previously [11]. Calibration curves were constructed by using weighted (1/*x*^2^) linear least squares regression. Data was collected with the MassLynx 4.0 software program. The data obtained in assays conducted COS-1 cells are depicted as % inhibition of substrate conversion, while the data obtained in H295R cells are expressed as absolute values (nM) ± SEM.

### 3.6. Statistical Analysis

All results are representative of two independent experiments, performed in triplicate, with the data being expressed as the mean ± SEM. After each assay a protein determination by the Pierce BCA method was performed in order to normalize steroid levels to protein concentrations. Statistical analyses were calculated with GraphPad Prism (version 5) software (GraphPad Software, San Diego, CA, USA) using a one-way ANOVA, followed by a Dunnett’s multiple comparison test. A value of *p* < 0.05 was considered to be statistically significant (* *p* < 0.05, ** *p* < 0.01, *** *p* < 0.001). 

## 4. Conclusions

This study included five of the major flavonoid compounds present in rooibos. These flavonoids, regardless of structural differences, all inhibited the activitiy of CYP17A1 and 3βHSD2, branch point enzymes in adrenal steroidogenesis. The dihydrochalcones and the flavone/flavonol compounds, however, showed marked differences in their inhibition of the activity of downstream enzymes, CYP21A2 and CYP11B1, expressed in COS-1 cells. In addition, these compounds also exhibited distinct differences in terms of specific substrates. It is clear that structural differences regarding the number and position of hydroxyl and glucose moieties, and structural flexibility, impact on the inhibitory effect of these flavonoids, possibly indicating different mechanisms. Rooibos extract, which contains a wide spectrum of flavonoid compounds, some of which are present at higher levels than was assayed, did not in all cases exhibit the same degree of inhibition. It is thus plausible that, of the array of compounds present in rooibos, some may inhibit while others may stimulate or have no effect at all. Overall, it is clear that, although the mechanism of inhibition is uncertain, the major rooibos flavonoid compounds assayed in this study significantly influence adrenal steroidogenic enzyme activities and steroid hormone levels.
